# Dimerization deficiency of enigmatic retinitis pigmentosa-linked rhodopsin mutants

**DOI:** 10.1038/ncomms12832

**Published:** 2016-10-03

**Authors:** Birgit Ploier, Lydia N. Caro, Takefumi Morizumi, Kalpana Pandey, Jillian N. Pearring, Michael A. Goren, Silvia C. Finnemann, Johannes Graumann, Vadim Y. Arshavsky, Jeremy S. Dittman, Oliver P. Ernst, Anant K. Menon

**Affiliations:** 1 Department of Biochemistry, Weill Cornell Medical College, 1300 York Avenue, New York, New York 10065, USA; 2 Department of Biochemistry, University of Toronto, Toronto, Ontario, Canada M5S 1A8; 3 Department of Ophthalmology, Duke University Medical Center, Durham, North Carolina 27710, USA; 4 Department of Biological Sciences, Center for Cancer, Genetic Diseases and Gene Regulation, Fordham University, Bronx, New York 10458, USA; 5 Weill Cornell Medicine—Qatar, Qatar Foundation, Education City P.O.Box 24144, Doha, State of Qatar; 6 Department of Pharmacology and Cancer Biology, Duke University Medical Center, Durham, North Carolina 27710, USA; 7 Department of Molecular Genetics, University of Toronto, Toronto, Ontario, Canada M5S 1A8

## Abstract

Retinitis pigmentosa (RP) is a blinding disease often associated with mutations in rhodopsin, a light-sensing G protein-coupled receptor and phospholipid scramblase. Most RP-associated mutations affect rhodopsin's activity or transport to disc membranes. Intriguingly, some mutations produce apparently normal rhodopsins that nevertheless cause disease. Here we show that three such enigmatic mutations—F45L, V209M and F220C—yield fully functional visual pigments that bind the 11-*cis* retinal chromophore, activate the G protein transducin, traffic to the light-sensitive photoreceptor compartment and scramble phospholipids. However, tests of scramblase activity show that unlike wild-type rhodopsin that functionally reconstitutes into liposomes as dimers or multimers, F45L, V209M and F220C rhodopsins behave as monomers. This result was confirmed in pull-down experiments. Our data suggest that the photoreceptor pathology associated with expression of these enigmatic RP-associated pigments arises from their unexpected inability to dimerize via transmembrane helices 1 and 5.

## Abstract (web summary)

Retinitis pigmentosa is often caused by mutations that affect the activity or transport of rhodopsin, but some mutations cause disease even though an apparently functional protein is produced. Here the authors show that three such enigmatic mutants retain scramblase activity but are unable to dimerize.

## Graphical abstract

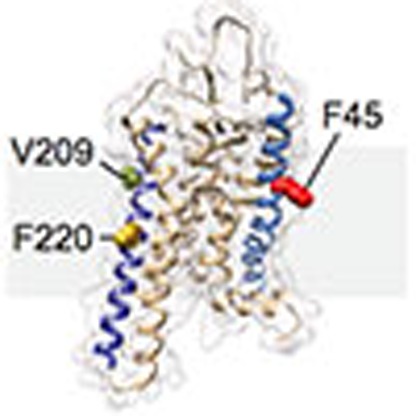

Retinitis pigmentosa (RP) is a retinal degenerative disease that is marked initially by a loss of rod photoreceptor cells responsible for dim light (night) vision^1^,^2^,^3^,^4^ and progresses eventually to complete blindness. RP affects over one million individuals worldwide (http://www.blindness.org/). One-third of all autosomal dominant RP cases are due to mutations in the visual pigment rhodopsin^5^,^6^,^7^, a prototypical G protein-coupled receptor (GPCR) consisting of the heptahelical apo-protein opsin linked to the vitamin A-derived chromophore, 11-*cis* retinal. The majority of RP mutations characterized so far result in rhodopsins that are deficient in their ability to fold correctly, bind retinal, activate the G protein transducin^8^ or traffic from biosynthetic membranes to the photoreceptor outer segment^9^. Intriguingly, a number of RP mutations appear to produce normally functioning visual pigments^1^,^10^. We now present a new insight into the mechanism by which these enigmatic mutations might cause disease.

In this study, we characterized three point mutations in opsin—F45L, V209M and F220C—which were identified in patients with clinical manifestations of RP^11^,^12^,^13^. These mutations occur at sites located on the surface of transmembrane (TM) helices 1 and 5 (Fig. 1; Ballesteros–Weinstein numbers^14^: 1.40 (F45), 5.44 (V209) and 5.55 (F220)). Experimental tests^8^,^15^,^16^ and *in-silico* analyses^1^,^10^ suggested that these proteins should function normally as visual pigments. We now show that these three mutants indeed share many critical properties with wild-type (WT) rhodopsin: they are correctly transported to rod outer segments and the plasma membrane when expressed in COS-7 cells, and are capable of binding and releasing retinal and activating transducin.

Our initial hypothesis to explain why these seemingly normal proteins cause disease was inspired by the recent demonstration that rhodopsin is a constitutively active phospholipid scramblase^17^,^18^,^19^. When reconstituted into large unilamellar vesicles, rhodopsin translocates common phospholipids rapidly across the membrane, independently of ATP, light and retinal^17^,^18^,^19^. We considered whether the enigmatic RP mutations might cause a deficit in rhodopsin's scramblase activity. We speculated that in the absence of normal scrambling, disc membrane homeostasis^19^ would be compromised, leading to disease pathology. However, we found that all three RP-associated rhodopsins have phospholipid scramblase activity similar to WT protein.

Although using scramblase activity to monitor detergent-mediated reconstitution of rhodopsin into large unilamellar vesicles we discovered remarkably that whereas WT rhodopsin inserts into the vesicles minimally as a dimer, each of the three mutants reconstitutes as a monomer. We confirmed this result with pull-down assays and used *in silico* approaches to show that the mutation sites lie at distinct dimerization interfaces. As these mutations do not affect scramblase activity, these data rule out a prevailing hypothesis^18^ that scrambling requires a rhodopsin dimer interface. Our results suggest that expression of the F45L, V209M and F220C RP-associated rhodopsins causes disease because of the inability of these proteins to dimerize via TM1 and TM5. Dimerization deficiency could have multiple consequences, including the inability to generate appropriate quaternary structures in disc membranes that may be required for disc assembly and maintenance^20^. Our results have broad significance for understanding the mechanistic basis of ubiquitously observed dimerization and multimerization of the large class of rhodopsin-like GPCRs, which has been proposed to regulate the ability of these proteins to transduce signals and is considered a target for pharmacological intervention^21^.

## Results

### F45L V209M and F220C rhodopsins are normal visual pigments

**Figure 1:**
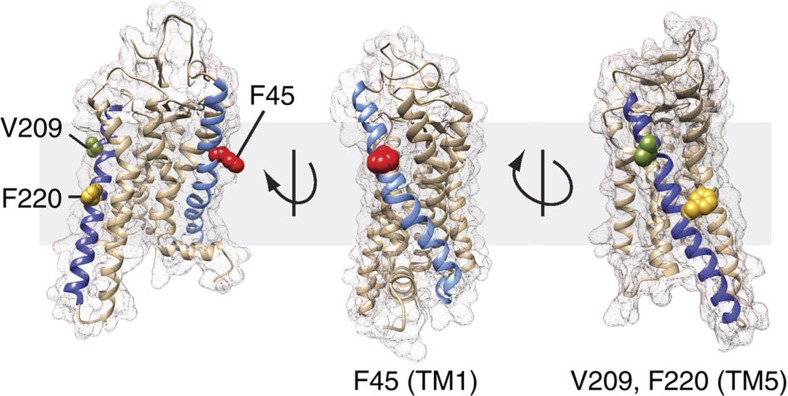
Rhodopsin structure showing sites of RP mutations. Three views of rhodopsin (PDB accession 4J4Q) are shown in the context of a lipid bilayer (light grey slab); TM helices 1 (TM1, light blue) and 5 (TM5, dark blue) are highlighted. The amino acids that are affected in the RP mutants under study are shown as space-filling CPK representations (F45 (red), V209 (green) and F220 (gold)). Ballesteros–Weinstein numbers^14^ corresponding to the affected residues are as follows: 1.40 (F45), 5.44 (V209) and 5.55 (F220).

**Figure 2:**
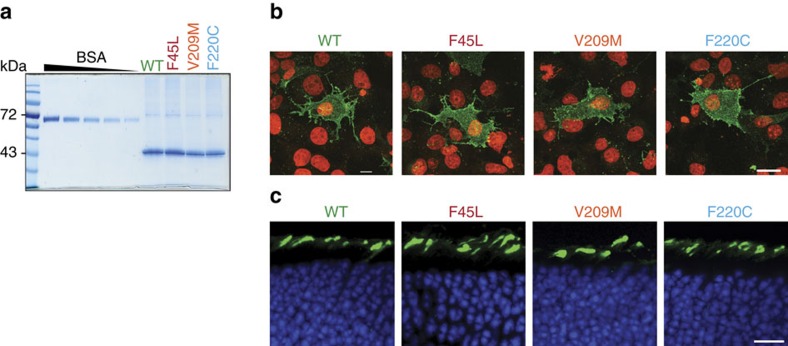
Expression of RP mutants. (**a**) SDS–PAGE analysis of purified WT opsin and RP-associated mutants. The gel was visualized by Coomassie staining. BSA (1 μg–200 ng) was run alongside and used for quantification purposes. (**b**) Fluorescence micrographs of non-permeabilized COS-7 cells expressing WT and mutant opsin as indicated. Cell surface opsin (green) was detected with the Ret-P1 antibody that recognizes an N-terminal (extracellular) epitope; nuclei (red) were stained with 4,6-diamidino-2-phenylindole (DAPI). Scale bar, 20 μm. (**c**) Rhodopsin immunostaining (green) in cross-sections of rhodopsin knockout mouse retinas transfected with WT, F45L, V209M and F220C rhodopsin constructs, respectively. Nuclei are stained with Hoechst (blue). Scale bar, 10 μm.

We introduced the F45L, V209M and F220C mutations (Fig. 1) into a thermostable N2C/D282C version of opsin^22^, expressed the mutant proteins in HEK293S GnTI^−^ cells and purified them from a detergent extract of the cells by affinity chromatography. Purified opsin mutants were obtained in yields comparable to that of the WT thermostable protein (Fig. 2a). We also used the same cell system to express green fluorescent protein-tagged versions of the F45L and F220C mutants. Analysis by fluorescence size-exclusion chromatography^23^ revealed a sharp, symmetrical profile for each mutant, identical to the profile obtained for WT opsin (Supplementary Fig. 1). These results indicate that the F45L and F220C opsin mutants are as monodisperse as the WT protein.

To determine whether the F45L, V209M and F220C rhodopsins are processed normally in biosynthetic membranes and exported from them, we used indirect immunofluorescence microscopy. The mutants were expressed in COS-7 cells and their appearance at the plasma membrane was probed with Ret-P1, an antibody that recognizes an epitope near the exoplasmic amino terminus of the protein. As shown in Fig. 2b, all three mutants could be detected at the surface of non-permeabilized cells, similar to WT rhodopsin, indicating that they are normally processed and delivered to the plasma membrane^8^,^15^,^16^. We also expressed WT and the three rhodopsin mutants in rod photoreceptors using the technique of *in vivo* electroporation^24^,^25^. In these experiments, we used rhodopsin knockout mice to avoid any possibility that the presence of endogenous rhodopsin could influence intracellular processing and trafficking of the mutant rhodopsins. Similar to WT rhodopsin, all three mutants showed normal outer segment localization when retinas were examined on postnatal day 21 (Fig. 2c; it is noteworthy that only a subset of rods is typically transfected by this technique). Taken together, these results demonstrate that the F45L, V209M and F220C mutations do not affect intracellular trafficking of rhodopsin.

**Figure 3:**
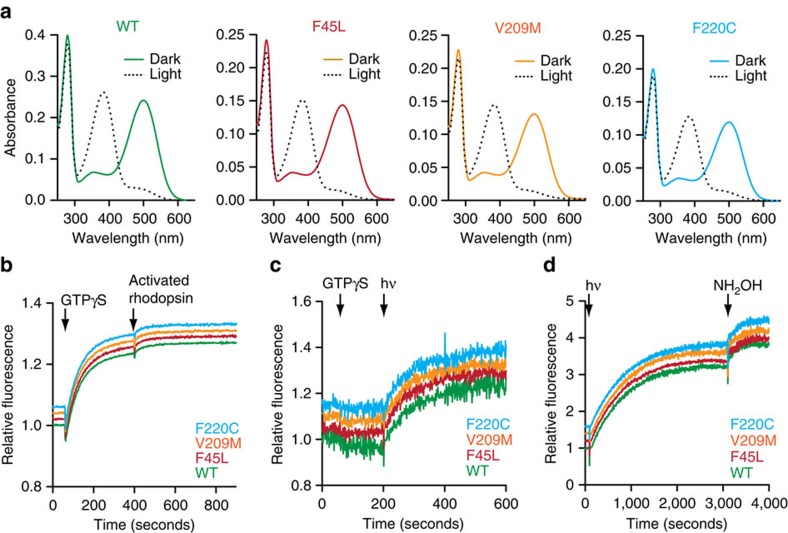
RP mutants are functional visual pigments. (**a**) Ultraviolet-visible spectra of purified WT rhodopsin and RP-linked rhodopsin mutants. The solid traces correspond to spectra measured in the dark, whereas the dashed traces represent spectra obtained after illuminating the samples for 90 s with >515 nm light. (**b**) G protein activation assay. The traces show the time course of intrinsic fluorescence of the G protein transducin. Fluorescence increases on GDP–GTP exchange in the Gα subunit, corresponding to its activation. The sample containing transducin and a catalytic amount of rhodopsin was illuminated for 60 s, starting at *t*=0 s. Nucleotide exchange was initiated by addition of GTPγS (arrrow). At *t*=400 s, an excess amount of light-activated WT rhodopsin was added to test for the completeness of transducin activation. (**c**) Transducin fluorescence measurement as in **b**, conducted before and after light activation of rhodopsin (arrow hν). After adding GTPγS at *t*=60 s, the intrinsic fluorescence of transducin did not change due to lack of active rhodopsin. At *t*=200 s, the sample was illuminated (arrow) to activate rhodopsin and initiate GDP–GTP exchange. (**d**) Retinal release. The traces show the intrinsic fluorescence increase on retinal Schiff base hydrolysis and retinal release from light-activated rhodopsin. The reaction was initiated by illumination of rhodopsin at *t*=90 s. At *t*=3,100 s, NH_2_OH was added to hydrolyse the remaining retinal Schiff base, thereby facilitating the release of any remaining retinal. For **b**–**d**, the fluorescence was normalized to the level at the start of the recording and the traces in each panel are vertically displaced for clarity (the displacements are 0.02 units in **b**, 0.05 units in **c** and 0.2 units in **d**).

We next asked whether the mutant proteins are capable of functioning normally as visual pigments *in vitro*. As shown in Fig. 3a, all three mutants showed WT-like ultraviolet-visible spectra when reconstituted with 11 *cis*-retinal and purified in the dark (Fig. 3a, solid traces). On illumination, they were all fully converted to a 380 nm-absorbing photoproduct, indicating normal formation of metarhodopsin II, the active state of rhodopsin (Fig. 3a, dashed traces). Figure 3b,c show GDP–GTP nucleotide exchange in the G protein transducin catalysed by light-activated rhodopsin. Nucleotide exchange was monitored by an increase of intrinsic transducin fluorescence on uptake of the non-hydrolysable GTP analogue GTPγS by the transducin α-subunit 26,27. For these experiments, a mixture of rhodopsin (WT or mutant) and transducin was illuminated for 60 s (starting at *t*=0 s) before adding GTPγS and observing the change in fluorescence. The RP-associated mutants catalysed nucleotide exchange exactly as the WT protein (in all cases GDP–GTPγS exchange was nearly complete at *t*=400 s as the addition of an aliquot of photoactivated WT rhodopsin did not significantly increase the response amplitude (Fig. 3b)). The light dependence of nucleotide-exchange catalysis was confirmed by carrying out the same experiment in the dark (Fig. 3c). Here, the intensity of excitation light in the fluorimeter was kept low, to prevent inadvertent rhodopsin activation; hence, the fluorescence traces are noisier than those shown in Fig. 3b. No nucleotide exchange was observed on adding GTPγS, until the sample was illuminated with bright light (indicated by hν, Fig. 3b), showing that rhodopsin-catalysed nucleotide exchange on transducin was light dependent in all cases. Finally, similar to WT rhodopsin, the mutants released retinal following rhodopsin bleaching as monitored by changes in the intrinsic tryptophan fluorescence of rhodopsin (Fig. 3d)^28^. We conclude that the F45L, V209M and F220C RP-associated rhodopsins are fully functional as visual pigments.

### F45L V209M and F220C opsins have normal scramblase activity

**Figure 4:**
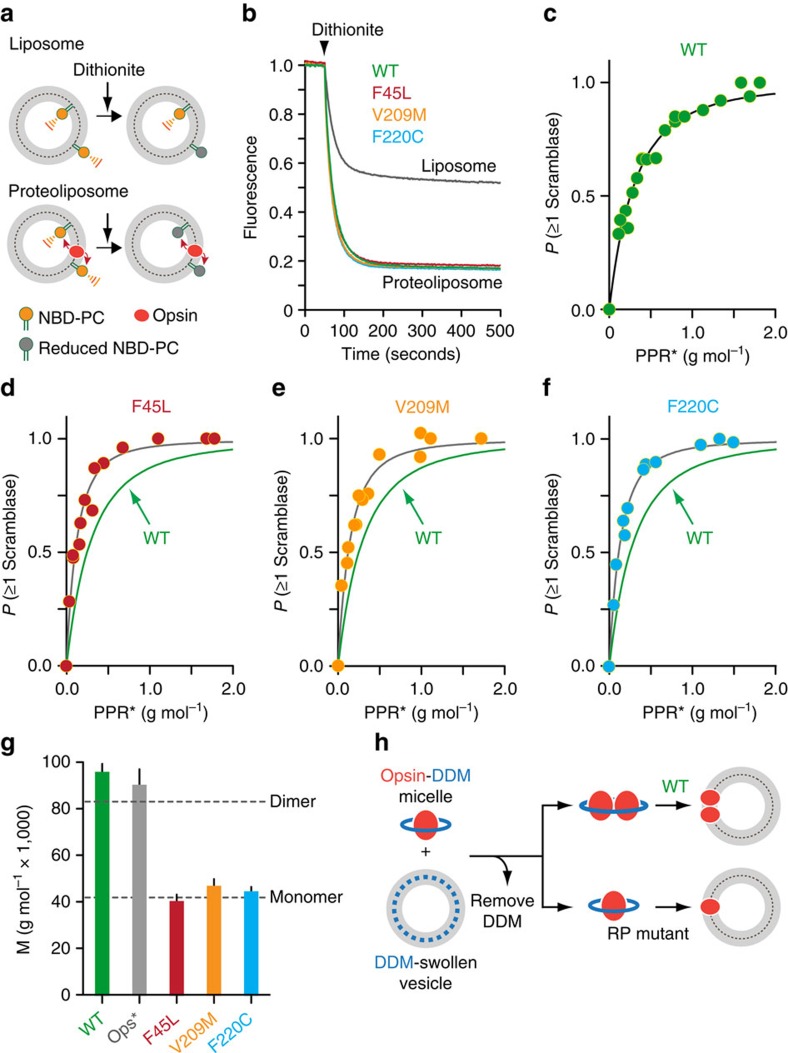
Scramblase activity of RP mutants. (**a**) Schematic representation of the scramblase activity assay. Details may be found in the text and Supplementary Note 1. (**b**) Fluorescence traces obtained on adding dithionite to NBD-PC-containing protein-free liposomes (grey trace) and proteoliposomes reconstituted with different opsins (coloured traces) at a high PPR (∼1 g mol^−1^). Each trace represents the mean of three independent experiments; the variation between experiments was negligible and is contained within the thickness of the lines. (**c**) Scramblase activity as a function of the amount of WT opsin reconstituted. Scramblase assays were performed for 400 s with vesicles reconstituted at different PPRs (PPR*, in units of grams of protein per mole of phospholipid, obtained by scaling the measured PPR as described in ‘Methods'). The plot represents the dependency of *p*(≥1) scramblase (the probability of a vesicle having at least one scramblase) on the PPR*. The line represents the data fit calculated as described in ‘Methods' and in Supplementary Notes 2 and 3. The data are from five independent protein preparations. (**d**) As in **c**, for the F45L mutant. The trace for the WT sample is from **c**. (**e**) As in **d**, for the V209M mutant. (**f**) As in **d**, for the F220C mutant. Data shown in **d**–**f** are from three independent protein preparations in each case. (**g**) Molar mass (M) of the functionally reconstituted scramblase (mean±s.e., corresponding to Supplementary Table 2) derived from fits of the *p*(≥1) scramblase versus PPR* data for WT opsin, Ops* and the RP-associated mutants. Dashed lines indicate the molecular weights of opsin monomer and dimer. (**h**) Schematic illustration of reconstitution modes of WT and RP mutant opsins. All opsins are purified in DDM as monomers. Opsin-DDM micelles are combined with DDM-destabilized vesicles and detergent withdrawal is initiated by adding SM2 BioBeads. When detergent is withdrawn WT opsin dimerizes (or multimerizes), while still within DDM micelles. As DDM continues to be removed from the system, the dimers insert into the available vesicles. For opsin RP mutants, dimerization does not occur and the proteins enter vesicles directly, as monomers.

We next tested whether the RP-associated mutants are able to scramble phospholipids. To measure scramblase activity, we reconstituted purified proteins into preformed large unilamellar vesicles together with the fluorescent phospholipid reporter 1-palmitoyl-2-(6-[7-nitro-2-1,3-benzoxadiazole-4-yl)amino]hexanoyl)-*sn*-glycero-3-phosphatidylcholine (NBD-PC). In parallel, we generated NBD-PC-containing protein-free vesicles. The reconstitution procedure results in NBD-PC distributing equally between the inner and outer leaflets of the vesicle membrane^18^. Scramblase activity was assayed using a previously described method (Fig. 4a)^17^,^18^,^29^,^30^,^31^ in which the vesicles are treated with dithionite, a reducing agent that eliminates NBD fluorescence by chemically altering the nitro group in NBD to an amino group^32^. Dithionite is negatively charged and cannot readily cross membranes^32^, including membranes containing rhodopsin^18^, and thus it acts only on NBD-PC molecules located in the outer leaflet of the vesicles (Fig. 4a). Thus, when dithionite is added to symmetrically labelled protein-free vesicles in which the rate of phospholipid scrambling is negligible on the time scale of our experiments^33^, ∼50% of the fluorescence is expected to be lost as NBD-PC molecules in the outer leaflet are reduced, whereas those in the inner leaflet are protected. However, in vesicles containing a functional scramblase, all fluorescence is predicted to be lost on adding dithionite, as scrambling enables NBD-PC molecules situated in the inner leaflet to gain access to the outer leaflet (Fig. 4a).

To determine whether the F45L, V209M and F220C opsins are able to scramble phospholipids, we reconstituted them into vesicles at a high protein-to-phospholipid ratio (PPR), ∼1 g protein per mole phospholipid, corresponding roughly to ten opsin monomers or five opsin dimers per vesicle. This was sufficient to ensure statistically that the majority of vesicles are reconstituted with at least one opsin molecule. Dithionite addition to protein-free liposomes resulted in ∼45% reduction of the fluorescence signal, whereas treatment of proteoliposomes reconstituted with the mutant proteins yielded a much greater extent of fluorescence reduction, ∼85% (Fig. 4b and Supplementary Fig. 2C–E). Identical results were obtained for vesicles containing WT opsin (Fig. 4b and Supplementary Fig. 2A) or Ops*, an active form of opsin which is similar to metarhodopsin II, which we previously showed was functional as a scramblase 18 (Supplementary Fig. 2B). The kinetics of fluorescence loss could be approximated in all cases by a mono-exponential decay function with *t*_1/2_ ∼15 s (Supplementary Figs 2 and 3, Supplementary Table 1 and Supplementary Note 1), except a small deviation for F45L where we obtained *t*_1/2_ of ∼20 s (Supplementary Fig. 3 and Supplementary Table 1). As the *t*_1/2_ values are comparable between protein-free liposomes and proteoliposomes (Supplementary Table 1), the rate of lipid scrambling (we estimate >10^4^ lipids per opsin per second^17^,^18^) cannot be separated from the rate at which the NBD fluorophore is reduced by dithionite and thus the assay provides an end-point measurement. Despite carrying out reconstitutions such that each vesicle should contain multiple copies of opsin on average, we never observed a fluorescence reduction greater than ∼85%. This is consistent with previously published results^17^,^18^ and suggests that some vesicles in the population cannot be reconstituted with protein. This point is discussed in Supplementary Note 2 (refs 19, 34, 35, 36, 37, 38). Taken together, our results indicate that all three RP-associated mutants are competent to scramble phospholipids, and that their scramblase activity is similar to that of WT rhodopsin.

### F45L V209M and F220C opsins reconstitute as monomers

We noticed that reconstitution of relatively small amounts of the RP-associated opsin mutants compared with WT opsin or Ops* yielded a high extent of fluorescence reduction in the scramblase assay. As the efficiency of reconstitution was the same in all cases (∼70% recovery of both protein and phospholipid in the reconstituted vesicles, see Methods), we considered an alternative explanation for this phenomenon. We previously reported that purified WT opsin and Ops* are monomeric in dodecyl-β-D-maltoside (DDM) detergent micelles^18^,^39^, but they self-associate as the detergent is gradually removed during the reconstitution procedure. Thus, both opsin and Ops* reconstitute into vesicles minimally as dimers (schematically illustrated in Fig. 4h, ‘WT')^18^. This was verified by chemical cross-linking experiments^18^. We considered the possibility that the mutant opsins do not dimerize during detergent removal and therefore functionally reconstitute into vesicles as monomers (Fig. 4h, ‘RP mutant'). Assuming that the monomeric proteins are functional as scramblases, this would explain why a smaller amount of mutant compared with WT opsin molecules is sufficient to produce a population of vesicles with scramblase activity.

To test this hypothesis, we measured the extent of scrambling in a series of proteoliposomes prepared with different PPRs. The extent of scrambling is a direct measure of the fraction of vesicles that is equipped with at least one functional scramblase and is equivalent to the probability, *p*(≥1 scramblase), which a particular vesicle in the population acquires at least one functional scramblase during reconstitution. This probability increases as a function of the PPR value and can be described by a simple model based on Poisson statistics^17^,^18^. We now advanced this model to take into account the measured size distribution of the vesicle population (Supplementary Fig. 4); we also accounted for the pool of vesicles that cannot be reconstituted with protein (Supplementary Notes 2 and 3).

We reconstituted a range of protein amounts (100 ng to 5 μg) into a fixed quantity of large unilamellar phospholipid vesicles (4 μmol total lipid) and measured the extent of scrambling. The dependence of *p*(≥1 scramblase) on PPR* (a scaled version of PPR that corrects for the presence of the pool of vesicles that cannot be reconstituted with protein (Supplementary Notes 2 and 3)) for WT opsin is shown in Fig. 4c (a larger data set is shown in Supplementary Fig. 5A). The line through the data points corresponds to our analytical model (Supplementary Note 3). The molar mass of the functional scramblase calculated from the associated fit constant was equal to 96,500±3,240 g mol^−1^ (± indicates s.e. associated with the fit; Fig. 4g and Supplementary Table 2). This replicates our previous findings^18^ and indicates that WT opsin reconstitutes as a mixture of dimers (molecular weight 83,400 Da) and higher-order multimers. A similar analysis for Ops* (Supplementary Fig. 5B) yielded the molar mass of functionally reconstituted scramblase to be 90,800±6,540 g mol^−1^ (± indicates s.e. associated with the fit; Fig. 4g and Supplementary Table 2), corresponding approximately to the mass of Ops* dimers, also as we reported previously^18^. For the three RP mutant opsins the outcome was dramatically different, in line with our hypothesis that the mutants fail to dimerize. In each case the plot of *p*(≥1 scramblase) versus PPR* rose more steeply than the plot for WT opsin before saturating (Fig. 4d–f) and the deduced molar mass of the functionally reconstituted proteins fell in the range of 40,600–47,200 g mol^−1^ (Fig. 4g and Supplementary Table 2), corresponding to the molar mass of an opsin monomer (41,700 g mol^−1^). Therefore, we conclude that each of the three RP-associated mutations individually affects the ability of opsin to dimerize during the detergent withdrawal step of reconstitution, resulting in the functional reconstitution of monomers (Fig. 4h, ‘RP mutant'). Importantly, these data clearly demonstrate that an opsin monomer is active as a scramblase.

### Pull-down assays show that RP mutant opsins do not dimerize

**Figure 5:**
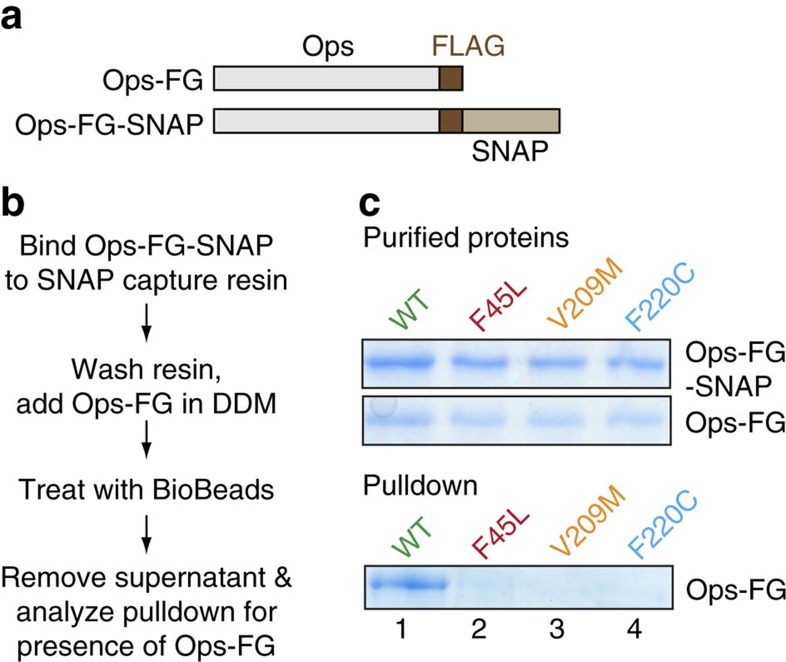
Pull-down experiments. (**a**) Schematic representation of the fusion proteins. (**b**) Protocol for the pull-down experiment. (**c**) Purified Ops-FG-SNAP and Ops-FG proteins are shown in the SDS–PAGE/Coomassie-stained gel panels labelled ‘Purified proteins' (5 μl samples were loaded from ∼100 ng μl^−1^ stocks; it is noteworthy that the Ops-FG-SNAP constructs stain more intensely than the Ops-FG proteins). The pull-down experiment was carried out as described in ‘Methods' and as outlined in **b**. Ops-FG-SNAP protein (2 μg) corresponding to either WT or RP-associated mutants was covalently pre-bound to SNAP capture resin in 0.1% (w/v) DDM and the resin was washed before adding cognate Ops-FG protein (2 μg in 0.1% (w/v) DDM). The mixture was treated with Biobeads as described in ‘Methods' before removing the supernatant, washing the resin and analysing the resulting pulldowns by SDS–PAGE/Coomassie staining after elution from the resin with Laemmli buffer (panel labelled ‘Pull-down'). The pull-down gel is representative of four independent experiments. Entire gels corresponding to the panels ‘Purified proteins' and ‘Pull-down' are shown in Supplementary Fig. 6C,D.

Chemical cross-linking reveals the presence of dimers and higher-order oligomers of rhodopsin in bovine photoreceptor discs treated with a low amount of detergent. No cross-linked products are seen if the discs are first exposed to high quantities of detergent, a condition where the disc is solubilized and rhodopsin is known to be monomeric^18^,^40^. Using a similar approach we previously showed that DDM-solubilized monomeric rhodopsin can be cross-linked into dimers when the detergent level is reduced by BioBeads treatment, as would occur in the early stages of proteoliposome reconstitution^18^. To probe dimerization of WT and RP-associated mutant opsins under similar conditions of detergent lowering that mimic proteoliposome reconstitution, we opted for pull-down assays instead of chemical cross-linking. For these experiments, we generated an Ops-FG-SNAP construct (Fig. 5a), in which a ∼19 kDa SNAP tag was fused to the carboxy-terminus of Ops-FG (Fig. 5a), the FLAG-tagged opsin construct that we used for our scramblase assays. RP-associated mutants corresponding to Ops-FG-SNAP and Ops-FG were also generated. All constructs were expressed in HEK293S GnTI^−^ cells and purified in 0.1% (w/v) DDM via their FLAG tag (Fig. 5c, ‘Purified proteins').

The pull-down experiment is described in Fig. 5b. Ops-FG-SNAP proteins in 0.1% (w/v) DDM were covalently bound to SNAP capture resin and the resin was washed in the same buffer before incubation with the cognate Ops-FG proteins, also in 0.1% (w/v) DDM. Samples were then treated with BioBeads to reduce DDM levels. Next, the SNAP-resin was spun down, separated from the BioBeads and washed with detergent-free buffer. Ops-FG proteins interacting with the covalently bound Ops-FG-SNAP were released from the SNAP-resin using Laemmli buffer and analysed by SDS–PAGE/Coomassie staining.

No Ops-FG proteins were pulled down in samples that were not treated with BioBeads (Supplementary Fig. 6A,B and Supplementary Note 4) consistent with both WT and RP-associated mutant opsins existing as monomers in high detergent, as expected. However, as shown in Fig. 5c, WT Ops-FG-SNAP is able to pull down its cognate Ops-FG partner after BioBead treatment (Fig. 5c, ‘Pull-down', lane 1), whereas the mutant proteins are not (Fig. 5c, ‘Pull-down', lanes 2–4). This result reinforces our conclusion that, whereas WT opsin dimerizes/multimerizes during detergent removal, RP-associated mutants remain monomeric (Fig. 4h).

### F45 V209 and F220 are located at dimer interfaces

The three RP-associated mutations analysed in this study correspond to residues located at the surface of TM1 (F45) and TM5 (V209, F220; Fig. 1). As these helices have been implicated in rhodopsin dimer formation^40^,^41^,^42^,^43^, we investigated whether F45, V209 and F220 are physically situated at predicted dimer interaction interfaces where mutations could plausibly disrupt dimer formation.

**Figure 6:**
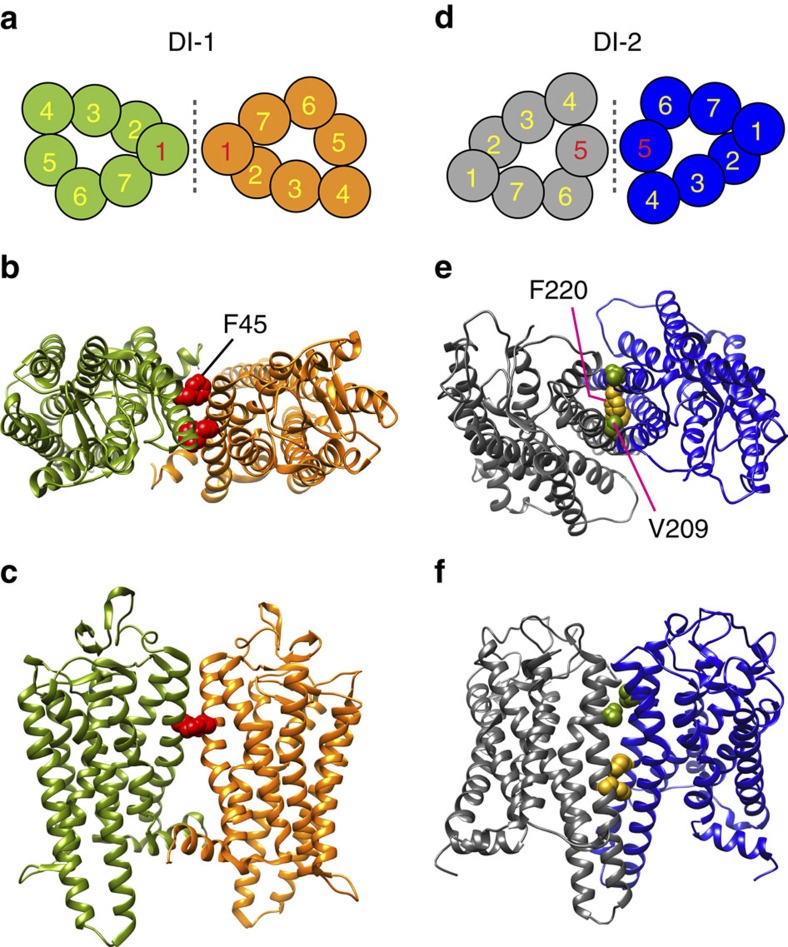
Rhodopsin dimer models. (**a**,**d**) Cross-sectional view of two distinct dimer interfaces (DI-1 and DI-2) in rhodopsin. The numbered circles correspond to individual helices in the TM helical bundle of each uniquely coloured monomer. (**b**,**c**) Top and side views of the interaction interface DI-1 formed by TM1-helix 8 in the opsin crystal structure PDB accession 3CAP. F45 is shown as a CPK model in red. (**e**,**f**) Top and side views of the interaction interface DI-2 in which TM5 plays a role. This structural model, obtained by docking the opsin structure (PDB accession 4J4Q) using the HADDOCK webserver, corresponds to cluster-6 in Supplementary Fig. 9. V209 (green) and F220 (gold) are shown as CPK models.

The opsin crystal structure (PDB accession 3CAP)^44^ shows an opsin dimer in the active Ops* conformation with TM1 at a potential dimer interface (DI-1, Fig. 6a–c). Inspection of this structure revealed that F45 occupies a critical position at the interface (Fig. 6b,c), contributing to ∼38% of the total in-membrane interactions provided by TM1 (calculated from the interaction interface generated by PDBsum Generate^45^). Whereas the crystal structure indicates that F45 interacts asymmetrically with alanine and leucine residues on the neighbouring chain, molecular dynamics studies reveal additional interactions at the TM1–TM1 interface where F45 is located^41^,^46^. Thus, it seems plausible that the substitution of a bulky, exposed aromatic phenylalanine residue with a relatively small aliphatic leucine in the F45L mutant would disrupt the interaction network of the TM1-based dimerization interface.

To understand the effects of V209M and F220C mutations on opsin dimerization, we generated TM5-based dimer models via docking experiments (dimer interface DI-2; Fig. 6d–f and Supplementary Note 5). This *in-silico* approach was necessary, because explicit structural descriptions of dimers in which TM5 is located proximate to the dimer interface are restricted to non-physiological, anti-parallel dimers seen only in crystal structures (Supplementary Fig. 7). We carried out two different docking experiments, focusing on interfaces involving TM4/TM5 and TM5/TM6, respectively (see Supplementary Information). A representative dimer model from the TM4/TM5-based docking experiment is shown in Supplementary Fig. 8. As both V209 and F220 are positioned away from the dimer interface region in this structure, this model was not considered further. For the TM5/TM6-based docking experiment (Supplementary Fig. 9), we identified dimers, comprising opsins oriented in parallel, in which TM5 was located in the interfacial region. Here, both V209 and F220 are directly situated within DI-2 (Fig. 6e,f).

These analyses rationalize our experimental findings that the RP-associated mutants behave as monomers. Thus, all three residues that are affected in these mutants occupy positions on the protein where they are likely to be directly involved in dimer formation.

## Discussion

The discovery of molecular mechanisms for unexplained forms of rhodopsin-related autosomal dominant RP is of considerable interest for both fundamental understanding of molecular mechanisms of vision and the design of new therapeutics^6^. Here we demonstrate that three enigmatic RP-linked rhodopsin mutants (F45L, V209M and F220C) are fully active as visual pigments and phospholipid scramblases, but nevertheless display a common unexpected structural defect that may explain why they cause disease. We show that these mutants are not able to dimerize during detergent-mediated reconstitution and speculate that this defect manifests itself in the dysregulation of rhodopsin organization in disc membranes, potentially affecting many aspects of disc biogenesis and maintenance, ultimately leading to disease pathology.

The inability of the RP-associated rhodopsin mutants to dimerize during detergent-mediated reconstitution (Fig. 4h) is surprising, as our results and those of others clearly show that there is more than one way in which rhodopsin monomers can interact to form dimers (Fig. 6)^40^,^42^,^43^ and each single mutation would be expected to affect only one route of dimerization. Nevertheless, it appears that the probability of forming any dimer is reduced considerably if even one of the routes to dimer formation is compromised. Consistent with this intriguing result, a recent report indicated that synthetic peptides corresponding to TM1–TM4 are individually able to disrupt rhodopsin dimerization^42^. Using bioluminescence resonance energy transfer, the authors showed that rhodopsin dimerization in the plasma membrane of cultured cells could be severely impacted by incubating the cells with either synthetic peptides corresponding to TM1 or TM5 (ref. 42). Similar to our observations, this result appears counterintuitive, as a peptide targeting one interaction interface would not be expected to affect dimerization via an alternative interface. Thus, there appears to be intramolecular cross-talk in the way rhodopsin dimers are formed via distinct interactions involving different interfaces, such that a mutation in TM1 affects interactions that occur via TM5.

Rhodopsin is purified as a monomer in DDM and, as such, is able to activate the G protein transducin (Fig. 3b,c)^39^; furthermore, as a monomer it can also be phosphorylated by rhodopsin kinase and bind arrestin^47^. Cross-linking experiments using DDM-solubilized native disc rhodopsin indicate that it dimerizes when detergent is withdrawn, as is the case for our reconstitution procedure^18^,^40^. We report similar conclusions with WT opsin using pull-down assays (Fig. 5). Dimerization provides a mechanism to obscure hydrophobic surfaces that would otherwise need to be shielded by detergent. However, the presence of vesicles during the detergent withdrawal step provides an alternate way to shield the hydrophobic surface of the protein from inappropriate exposure to water by insertion into the vesicle membrane. Thus, direct insertion of a monomer into vesicles competes with the pathway in which dimers are formed and subsequently inserted (Fig. 4h). In the case of the RP mutants under study, it is clear that direct monomer insertion is favoured. This may occur if the different dimer states are exchangeable. Thus, the inability to exchange between dimer states, because a specific state is not available as a result of an RP-associated mutation, may affect the lifetime of the dimer and favour the monomer. More work will be needed to understand this phenomenon at the molecular level.

An important result of our study is that a rhodopsin monomer is capable of scrambling phospholipids. As WT rhodopsin molecules insert into vesicles as dimers or higher-order structures^18^ (Fig. 4g,h), it was not previously possible to determine the nature of a minimal functional rhodopsin unit capable of scrambling phospholipids. Indeed, in previous work we proposed that interfaces between rhodopsin monomers might provide privileged sites that could be used to translocate lipids^18^. Although these interfaces may indeed contribute to the lipid translocation pathway, we now demonstrate that they are not necessary for scrambling. We speculate instead that the interplay between rhodopsin's structure and the surrounding membrane is necessary to promote scrambling. As other Class A GPCRs also have phospholipid scramblase activity^18^, these ideas have general significance for studies of GPCR dynamics in membranes. It remains a mechanistic challenge for the future to understand how rhodopsin/GPCR monomers are able to scramble phospholipids.

Another goal of future studies is to elucidate how the inability of rhodopsin to form dimers leads to RP pathology. Rhodopsin is not only a visual pigment but also a critical building block required for the formation of discs and rod outer segments. Thus, there are many potential ways to link dimerization deficiency to RP pathology. We highlight two possibilities. First, recent work showed that guanylate cyclase-1, the enzyme responsible for generating the cGMP second messenger during phototransduction, requires rhodopsin for its delivery to the outer segment and to maintain its stability in this compartment^25^. It is conceivable that this cargo-carrying function of rhodopsin cannot be efficiently accomplished by monomers, accounting for disease pathology. Second, rhodopsin-knockout mice do not elaborate outer segments and their photoreceptors eventually degenerate and are lost^48^. Expression of rhodopsin at half the normal level (from a single copy of the gene) allows normal outer segment development but here also the photoreceptors degenerate, albeit over a longer time period^49^. Conversely, overexpression of rhodopsin alters the structure of rod photoreceptors, preserving rhodopsin packing density^50^.

The structural basis for rhodopsin's role in disc and outer segment morphogenesis and maintenance is not fully understood^20^,^42^ but it is increasingly clear that rhodopsin molecules are highly organized in disc membranes. Thus, there are several lines of evidence that rhodopsin forms dimers in disc membranes, and that a further supramolecular organization probably exists^43^,^51^,^52^,^53^,^54^,^55^. Recent work using cryoelectron tomography suggested that the organization of rhodopsin in intact photoreceptors can be described by a four-tier hierarchy^20^ involving rhodopsin dimers, rows of dimers and pairs of rows that form tracks that are aligned parallel to the disc incisures. This hierarchical organization of rhodopsin was suggested to be important for maintaining disc architecture^20^. It is tempting to speculate that as the nucleating step of rhodopsin dimerization may not occur efficiently in discs containing the F45L, V209M and F220C proteins, the potentially physiologically important supramolecular organization of rhodopsins would be compromised with severe consequences for disc architecture and stability. More work will be needed to define clearly the mechanistic link between rhodopsin dimerization deficiency and photoreceptor degeneration.

## Methods

### Materials

POPG (1-palmitoyl-2-oleoyl-*sn*-glycero-3-phospho-(1'-rac-glycerol), POPC (1-palmitoyl-2-oleoyl-*sn*-glycero-3-phosphocholine) and NBD-PC were from Avanti Polar Lipids (Alabaster, AL, USA); Bio-Beads SM2 Adsorbent, Bio-Spin chromatography columns and Bio-Gel P-6 were from Bio-Rad (Hercules, CA, USA); Micro-Spin columns with screw caps were from Thermo Scientific (Rockford, IL, USA); 100 × protease inhibitor cocktail was from EMD Millipore (Billerica, MA, USA); DDM was from Anatrace (Maumee, OH, USA); anti-FLAG M2 agarose and 3 × FLAG peptide were from Sigma (St Louis, MO, USA); SNAP-capture resin was from New England Biolabs, Ipswich, MA, USA); PfuUltra II DNA polymerase was from Agilent (Santa Clara, CA, USA); QiaFilter Plasmid Maxi kit was from Qiagen (Valencia, CA, USA); DMEM, 100 × penicillin/streptomycin were from Invitrogen (Grand Island, NY, USA); heat-inactivated fetal bovine serum (FBS) was from Atlanta Biologicals (Lawrenceville, GA, USA); DMEM/F12 was from Wisent (Saint Bruno, QC, Canada); FBS and DPBS from Life Technologies (Waltham, MA, USA); doxycycline from Bio Basic (Markham, ON, Canada); blasticidin and puromycin from Bioshop Canada (Burlington, ON, Canada); Jetprime transfection reagent from Polyplus Transfections (Illkirch, France); and Miniprep kit from Qiagen.

### Construction of mammalian expression vectors

Point mutations in the synthetic bovine opsin gene^56^ containing the N2C and D282C mutations for enhanced thermostability^22^ and a 3 × FLAG tag sequence at the 3′-end of the rhodopsin gene (further referred to as ‘wild type')^18^,^22^ were accomplished by two-step overlap extension PCR. NotI/EcoRI-restricted PCR fragments were inserted into the pMT3 expression vector^57^. The F45L, V209M and F220C mutations were introduced by using the following primers: gene-specific forward primer 5′-gcagaattccaccatgtgcgtaccgaag-3′; gene-specific reverse primer 5′-gtatgcggccgctcacttgtcatcgtcatccttg-3′; gene-specific reverse primer for SNAP tag containing constructs 5′-gtatgcggccgctcatcccagacccggtttac-3′; mutagenic forward primer for F45L 5′-ctccatgctggccgcctacatgctcctgctgatcatgcttggcttc-3′, mutagenic reverse primer for F45L 5′-gaagccaagcatgatcagcaggagcatgtaggcggccagcatggcg-3′; mutagenic forward primer for V209M 5′-gtcgttcgtcatctacatgttcatggtccacttcatcatcccgct-3′, mutagenic reverse primer for V209M 5′-cagcgggatgatgaagtggaccatgaacatgtagatgacgaacgac-3′; and mutagenic forward primer for F220C 5′-ctgattgtcatctgcttctgctatggccag-3′, mutagenic reverse primer for F220C 5′-ctggccatagcagaagcagatgacaatcag-3′. The sequence of each of the expression vectors was verified at the Cornell University Life Sciences Core Laboratories Center.

### Cell culture and transfection

Unless stated otherwise, HEK293S GnTI^−^ cells were used for protein expression. The cells do not have *N*-acetyl-glucosaminyltransferase I (GnTI) activity and lack the ability to synthesize complex *N*-glycans^58^; consequently, opsin produced in these cells is homogeneously *N*-glycosylated. Cells were cultured at 37 °C, in a 5% CO_2_ atmosphere^58^. For transfection, cells were harvested in exponential growth phase by trypsinization and washed once with warm cytomix buffer (120 mM KCl, 0.15 mM CaCl_2_, 10 mM K_2_HPO_4_/KH_2_PO_4_ pH 7.6, 25 mM HEPES pH 7.6, 2 mM EGTA pH 7.6 and 5 mM MgCl_2_). The cells were then resuspended at a density of 10^7^ cells per ml in cytomix buffer supplemented with 2 mM ATP and 5 mM glutathione. Twenty-five micrograms of a mixture (4:1 wt/wt) of two plasmids, pMT3 (ref. 22; carrying the thermostable opsin gene or mutant derivatives) and pRSVTag^59^ (carrying a gene encoding SV40 large tumour (T) antigen to promote pMT3 replication), and 400 μl of cell suspension were added to an ice-cold cuvette, mixed carefully and exposed to a single pulse of 300 V with a capacitance of 900 μF using a BioRad gene pulser system. Immediately after electroporation, 1 ml of pre-warmed medium was added and the sample was transferred to a 100-mm dish and cultured for 48 h before harvesting.

### Immunopurification and quantification of opsin variants

Transfected cells were washed twice in PBS and harvested by scraping using a rubber policeman. The cells were used immediately or pelleted and stored at −20 °C. Opsin variants were purified by FLAG affinity chromatography^18^ and recovered in 0.1% (w/v) DDM, 50 mM HEPES pH 7.4 and 100 mM NaCl. The proteins were quantified by Coomassie staining after SDS–PAGE, using an in-gel BSA standard (for example, as in Fig. 2a). The average yield for all opsin variants was ∼6 μg per 10^7^ transfected cells.

### Liposome preparation

Unilamellar liposomes were prepared by resuspending a lipid film in buffer A (50 mM HEPES pH 7.4 and 100 mM NaCl) and subjecting it to freeze–thaw extrusion. The procedure was exactly as described by Goren *et al*.^18^, except that POPC and POPG were used instead of egg PC and egg PA, respectively. The final concentration of the liposomes was typically 4 mM phospholipid. Dynamic light scattering revealed a Gaussian distribution of vesicle sizes with a mean radius of 88 nm (s.d.=28 nm; Supplementary Fig. 4).

### Proteoliposome preparation

Preformed liposomes were destabilized with detergent, incubated with detergent-solubilized opsin and NBD phospholipids, before being treated with BioBeads SM2 to remove detergent and accomplish reconstitution^18^. Protein content of proteoliposomes was determined by quantitative western blotting using anti-FLAG antibody (Sigma; F3165; dilution 1:5,000; the secondary antibody was anti-mouse IgG, horseradish peroxidase-conjugated (Promega; W402B) used at 1:5,000 dilution) that compared the purified opsin starting material to reconstituted opsins across the PPR range tested (four different amounts of the proteoliposome sample of interest and five amounts of an opsin standard were loaded on the same gel for this determination). Phospholipid content of proteoliposomes was determined via a colorimetric assay using duplicate amounts of the proteoliposome sample, calibrated against inorganic phosphate standards^60^. The relative error in protein and phospholipid measurements (s.e. as a percentage of the mean) was 6.95% and 1.9%, respectively, resulting in a relative error in PPR of 7.2%. Protein recovery after reconstitution was 71.4±3.9% (mean±s.e.m., *n*=14) and phospholipid recovery after reconstitution was 69.4±1.3% (mean±s.e.m., *n*=14).

### Scramblase assays

Scramblase activity was measured as described in Supplementary Note 1 (ref. 17). Briefly, 50 μl of NBD-PC-containing liposomes or proteoliposomes were added to 1,950 μl of Buffer A in a stirred cuvette. The fluorescence intensity (excitation 470 nm, emission 530 nm) was recorded in a PTI fluorescence spectrometer for at least 50 s before adding 40 μl of 1 M sodium dithionite (freshly made in unbuffered 0.5 M Tris). Fluorescence was recorded for an additional 400–600 s. Data were sampled at a frequency of 1 Hz.

### Analysis of scramblase reconstitution

The analysis is described in detail in Supplementary Notes 2 and 3. Briefly, end-point fluorescence reduction data from scramblase activity assays (that is, extent of fluorescence reduction 400 s after adding dithionite) were obtained for proteoliposomes generated over a range of PPR (grams of protein per mole phospholipid) values. The data were transformed according to the following equation:


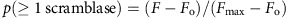


where *F* is the percentage fluorescence reduction for a particular sample 400 s after adding dithionite, *F*_o_ is the percentage reduction obtained with liposomes (45%, Supplementary Note 2), *F*_max_ is the maximum percentage reduction observed for samples with high PPR (82.5%; Supplementary Note 2) and *p*(≥1 scramblase) is the probability that a particular vesicle in the ensemble contains at least one functional scramblase. The dependence of *p*(≥1 scramblase) on PPR follows Poisson statistics. Taking into account that a fraction of the vesicles is refractory to reconstitution (Supplementary Note 2), and that the vesicles have a range of sizes that can be described by a Gaussian distribution (mean radius 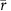
=88 nm and s.d. *σ*=28 nm (Supplementary Figure 4)), *p*(≥1 scramblase) can be written as (Supplementary Note 3, equation (4)):


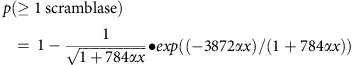


where *α* is a fit constant that is inversely proportional to *M*, the molar mass of the functional scramblase and *x* is the PPR*, derived from the measured PPR after taking into account the fraction of vesicles that is refractory to reconstitution (PPR*=PPR/0.65). Fitting data sets of *p*(≥1 scramblase) versus PPR* (Fig. 4c–f and Supplementary Fig. 5) yields *α* and hence the molar mass of the functionally reconstituted scramblase (Fig. 4g and Supplementary Table 2), for example, *α*=17.22 × 10^−4^ mol g^−1^ nm^−2^ corresponds to the functional reconstitution of opsin monomers (molar mass 41,700 g mol^−1^), whereas *α*=8.61 × 10^−4^ mol g^−1^ nm^−2^ corresponds to the functional reconstitution of opsin dimers.

### Immunofluorescence microscopy

COS-7 cells were seeded on 1.2 cm cover slips in 24-well plates at 5 × 10^4^ cells per cm^2^ in DMEM, 10% FBS 24 h before transfection with Lipofectamine 2000 (1.5 μl Lipofectamine and 1 μg plasmid DNA per well). Twenty-four hours after transfection, cells were fixed with 4% paraformaldehyde in PBS-CM (PBS, 0.1 mM CaCl_2_ and 1 mM MgCl_2_) for 20 min at room temperature before labelling with RET-P1 antibody (EMD Millipore; MAB5316) at 1 μg ml^−1^ and AlexaFluo594-conjugated donkey anti-mouse IgG secondary antibody (ThermoFisher/Invitrogen; A-21203) at 4 μg ml^−1^, both in PBS-CM. Nuclei were counterstained with 4,6-diamidino-2-phenylindole. Samples were mounted with Fluoromount G and imaged on a Leica TSP5 confocal microscopy system. Stacks of *x*–*y* scans imaged 0.25 μm apart were acquired for each field using constant acquisition parameters. The experiment was repeated three times independently and at least three fields were imaged for each plasmid in each experiment. Fields were compiled in Adobe Photoshop CS4. Brightness was adjusted identically for all fields.

### Expression and visualization of rhodopsins in mice

Rhodopsin knockout mice^49^ were handled following the protocols approved by the Institutional Animal Care and Use Committees of Duke University (registry number A212-12-08 and A011-14-01). Mice were housed under a 12/12 h light cycle. Retinal transfection of neonatal mice was performed using the *in vivo* electroporation technique^24^ with specific modifications described^25^. Following anaesthetization of neonatal mice on ice, the eyelid and sclera were punctured at the periphery of the eye using a 30-gauge needle. A blunt-end 32-gauge needle was advanced through the puncture wound until reaching the subretinal space and 0.3–0.5 μl of concentrated plasmid DNA (4 μg μl^−1^ of the construct of interest and 2 μg μl^−1^ mCherry to visualize transfected cells) was deposited. A tweezer-type electrode (BTX) was placed over the mouse's head with the positive electrode overlying the injected eye. Five 100–110 V pulses of 50 ms duration were applied using an ECM830 square pulse generator (BTX). Neonates were returned to their mother and allowed to develop until postnatal day 21 when mice were killed by CO_2_ inhalation followed by decapitation. A minimum of three positively expressing retinas were analysed for each DNA construct.

Mouse posterior eyecups were fixed for 1 h in 4% paraformaldehyde in mouse Ringer's solution, rinsed three times in PBS and embedded in 7.0% low-melt agarose (Sigma, A3038). One-hundred-micrometre cross-sections through the central retina were collected using a vibratome (Leica VT1200S), placed in 24-well plates and blocked in 5% goat serum and 0.5% Triton X-100 in PBS for 1 h at 22 °C. Sections were incubated in mouse monoclonal anti-rhodopsin 1D4 antibody (Abcam; ab5417) diluted 1:2,000 in blocking solution overnight at 4 °C, rinsed three times and incubated with 10 μg ml^−1^ Hoechst 33342 (ThermoFisher; H3569) and goat anti-mouse secondary antibodies conjugated with Alexa Fluor 488 (Invitrogen; A-11001) diluted 1:1,000 in blocking solution for 2 h at 22 °C. Sections were mounted with Fluoromount (Electron Microscopy Sciences) and cover-slipped.

### Spectral and functional characterization of rhodopsins

For spectroscopic characterization and G protein activation analyses, rhodopsin variants without the N2C/D282C thermostabilizing mutations were purified from inducible stable cell cultures using HEK293S GnTI^−^ cells and the piggyBac transposon-inducible system^65^, as previously described^61^,^62^. Cells were cultured in roller bottles until they reached confluence, treated with doxycycline (1 μg ml^−1^) to induce opsin expression and harvested 48 h after induction. Cell pellets were flash-frozen in liquid nitrogen and stored at −80 °C. Rhodopsin WT and RP-associated mutants were purified by affinity chromatography using 1D4-Sepharose (prepared from 1D4 antibody (www.rho1d4.com, BC, Canada) and CNBr-activated Sepharose 4B (GE Life Sciences, Marlborough, MA, USA)) 61.

Ultraviolet-visible spectroscopy was carried out using a Cary 60 UV (Agilent Technologies). Spectra (250–750 nm) were measured in the dark and after 90 s illumination using a >515 nm filter. The A280/A500 ratio was used to assess sample purity. Samples contained 0.03% (w/v) DDM and were at pH 6.0.

Transducin (*G*_t_) activation was measured by monitoring the change in tryptophan fluorescence as described using a SPEX Fluorolog 3 spectrofluorometer^26^,^39^. The experimental protocol was slightly modified as follows. Briefly, 1 nM WT rhodopsin, F45L, V209M or F220C were individually brought to 850 μl of measurement mixture containing 500 nM transducin^39^, 2 mM dithiothreitol, 130 mM NaCl, 1 mM MgCl_2_, 20 mM BTP pH 7.1 and 0.118 mM (0.005% w/v) DDM. The samples were added to a fluorescence cuvette with constant stirring and equilibrated at 20 °C for 4 min. For ‘light' measurements, the sample was illuminated with yellow light (*λ*>595 nm) for 60 s followed by baseline fluorescence recording for 60 s (excitation 300 nm, emission 345 nm). Transducin activation was then initiated by adding 200 μM GTPγS. After 400 s, 50 nM purified light-activated recombinant WT rhodopsin was added to test for the completeness of transducin activation. ‘Dark' measurements were performed similarly, except that the baseline was recorded for 60 s on the sample kept in the dark. Next, GTPγS was added to the sample and fluorescence recorded for another 140 s before GDP–GTP exchange (transducin activation) was triggered by illumination of the sample with yellow light 200 s after starting the fluorescence recording. The intensity of excitation light used in the fluorimeter was kept low, to avoid inadvertent activation of rhodopsin.

Retinal release from bleached rhodopsin was assayed as described^28^, by monitoring the change in rhodopsin tryptophan fluorescence.

### Pull-down assays

Ops-FG and Ops-FG-SNAP proteins (Fig. 5a) were purified by FLAG affinity chromatography as described above (‘Immunopurification and quantification of opsin variants'). For each pull-down assay, 50 μl of SNAP-capture resin was pre-equilibrated in buffer A containing 0.1% (w/v) DDM. Ops-FG-SNAP protein (2 μg) was incubated with the resin (final volume of solution: 100 μl) for 2 h, room temperature, with end-over-end mixing, resulting in a covalent bond between the SNAP tag and the SNAP capture resin. After two washes in buffer A (containing 0.1% (w/v) DDM), 2 μg of the respective purified Ops-FG protein was added in a final volume of 100 μl (0.1% (w/v) DDM). BioBeads SM-2 Resin (2.5 mg; the beads were washed as described for vesicle reconstitution and residual liquid was absorbed onto tissue paper to weigh out the beads reliably) was then added and the sample was incubated for 1 h at room temperature with end-over-end mixing. The SNAP resin was spun down, separated manually from the BioBeads and washed three times with buffer A. Any Ops-FG interacting with the covalently bound Ops-FG-SNAP-protein was eluted with Laemmli buffer and analysed by SDS–PAGE/Coomassie staining. Control experiments are described in Supplementary Note 4 and Supplementary Fig. 6A,B. Entire gels corresponding to Fig. 5c are shown in Supplementary Fig. 6C,D.

### Data availability

The authors declare that all data supporting the findings of this study are available within the article and its Supplementary Information files or are available from the corresponding author upon request.

## Additional information

**How to cite this article:** Ploier, B. *et al*. Dimerization deficiency of enigmatic retinitis pigmentosa-linked rhodopsin mutants. *Nat. Commun.* 7:12832 doi: 10.1038/ncomms12832 (2016).

## Supplementary Material

Supplementary Information Supplementary Figures 1-9, Supplementary Tables 1-2, Supplementary Notes 1-5, Supplementary References.
