# Supplementary material for: Dimerization deficiency of enigmatic retinitis pigmentosa-linked rhodopsin mutants
Source: Nat Commun. 2016 Oct 3;7:12832. doi: 10.1038/ncomms12832 (PMC5059438; doi:10.1038/ncomms12832)
Supplement: Supplementary Information — Supplementary Figures 1-9, Supplementary Tables 1-2, Supplementary Notes 1-5, Supplementary References. [file ncomms12832-s1.pdf]

Supplementary Figures

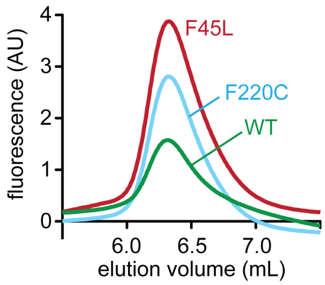

Supplementary Figure 1

FSEC of GFP-tagged opsins. The different opsins were expressed in HEK GnTI^{-} cells as fusion proteins with a C-terminal green fluorescent protein (GFP) tag, and extracted with lysis buffer (50 mM HEPES, pH 7.4, 100 mM NaCl, 0.1% (w/v) DDM). After centrifugation (50,000 x *g*, 10 min, 4°C) to remove insoluble material, the supernatant containing the opsin-GFP fusion protein was filtered using a Spin-X centrifuge tube filter before being analyzed by fluorescence size exclusion chromatography (FSEC). FSEC was performed on a Superdex 200 Increase 5/150 GL column (GE Healthcare Life Sciences) using a Shimadzu LC-20AD Prominence liquid chromatograph equipped with an RF-20A Prominence fluorescence detector (settings: excitation λ=395 nm, emission λ=507 nm). The column was washed with water and equilibrated with one column volume of lysis buffer before sample injection.

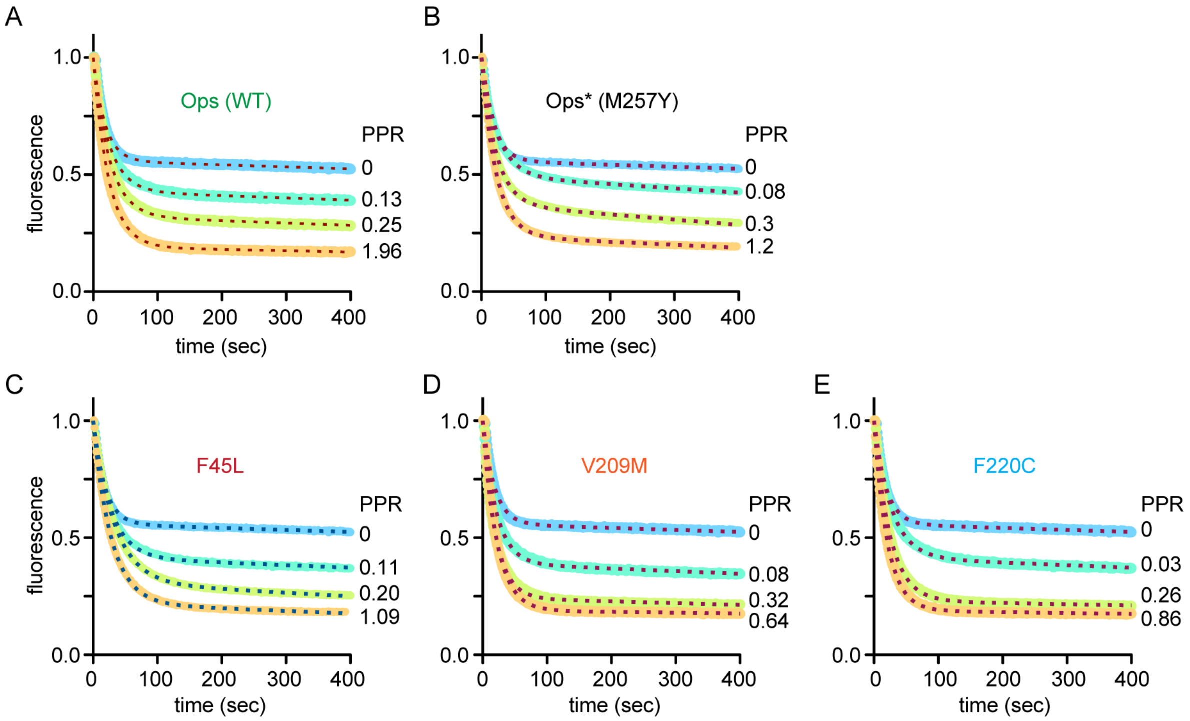

Supplementary Figure 2

Fluorescence traces from scramblase assays. Data are shown for thermostable (N2C/D282C) versions of Ops (WT) (A), Ops* (M257Y opsin) (B), F45L opsin (C), V209M opsin (D) and F220C opsin (E). NBD-phosphatidylcholine was the reporter lipid in all assays. Dithionite was added at time=0 sec. Individual traces are labeled according to the protein to phospholipid ratio of the vesicle preparation (PPR, in units of g protein per mol phospholipid, measured by quantitative immunoblotting for protein and a colorimetric assay for lipid phosphorus (see 'Methods')). The dotted line superimposed on each trace is a fit of the data performed as described in the text and Supplementary Table 1. A reference trace for protein-free liposomes (PPR = 0 g/mol) is included in each panel.

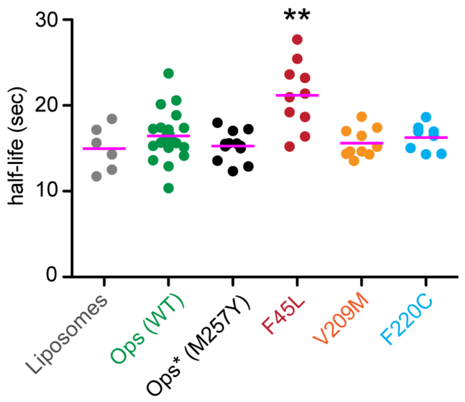

Supplementary Figure 3

Kinetics of fluorescence reduction in scramblase assays. The outcome of fits to individual fluorescence reduction traces (e.g., Supplementary Fig. 2) is shown, along with the mean of each data set (pink bars). The mean half-life for the F45L data set is significantly different (**p=0.001) from all the other means. The data correspond to Supplementary Table 1 (column 1).

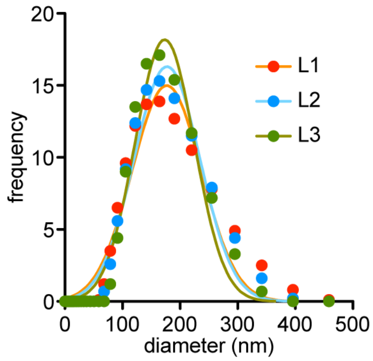

Supplementary Figure 4

Size distribution of vesicles. Three independent vesicle preparations (L1, L2, L3) were analyzed by dynamic light scattering using a Zetasizer Nano S instrument (Malvern Instruments Ltd, United Kingdom). Each data point shown corresponds to the mean of a triplicate measurement. The data were analyzed using a Gaussian distribution (lines on the graph), yielding an average vesicle radius $\bar{r}$ = 88 nm with a standard deviation σ = 28 nm.

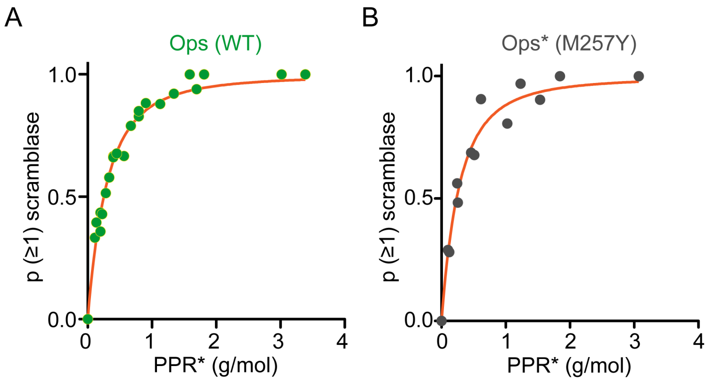

Supplementary Figure 5

p(≥1 scramblase) vs PPR for Ops(WT) and Ops*. End-point fluorescence reduction data were transformed to obtain plots of p(≥1 scramblase) versus PPR*. The data were fit to equation 4 (orange lines). The fit constant α and the deduced molar mass of the functional scramblase are tabulated in Supplementary Table 2. The data are from 5 (Ops(WT)) and 2 (Ops*) independent protein preparations.

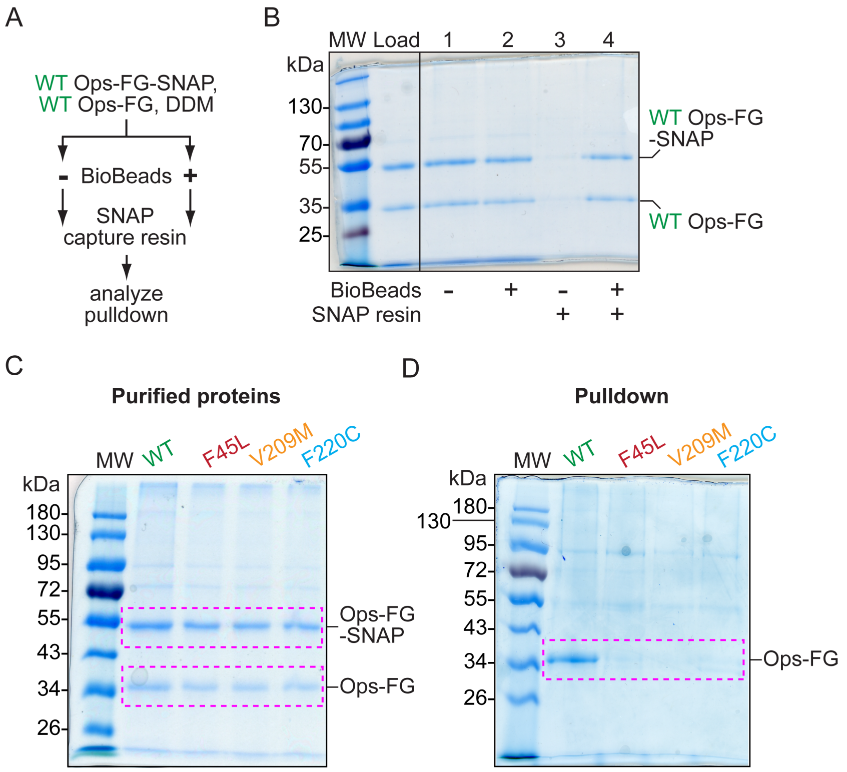

Supplementary Figure 6

Pulldown assays. A, B. Control experiments with WT opsin as described in Supplementary Note 4. Note that a slightly different protocol is used for the pull-down assay described in the main paper where Ops-FG-SNAP is pre-bound to SNAP capture resin before incubating the sample with Ops-FG (compare protocol shown in Fig. 5B of the main paper with panel A shown here). **C, D.** The Coomassie-stained SDS-PAGE gel panels shown in Fig. 5C of the main paper were taken from the gels shown here in their entirety. The panels excised for the main paper are boxed. MW, molecular weight markers.

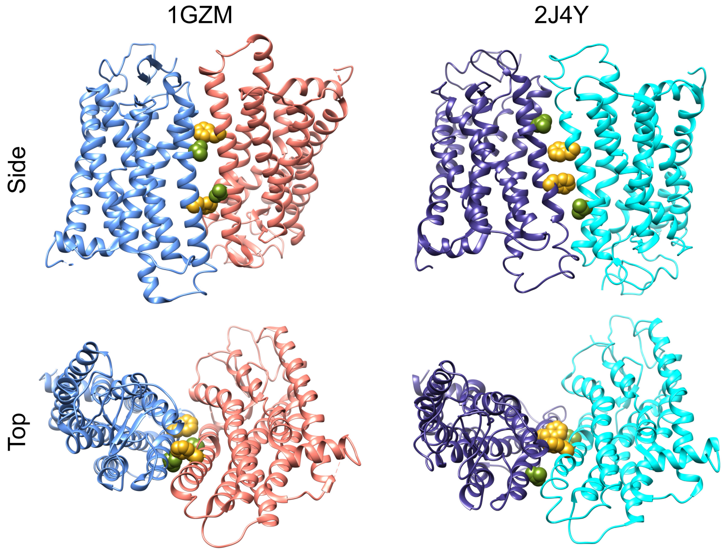

Supplementary Figure 7

Crystal structures of antiparallel rhodopsin dimers where TM5 is positioned proximate to the interface between monomers. Side and top views of PDB 1GZM and 2J4Y are shown (PDB 2J4Y corresponds to the thermostable N2C/D282C rhodopsin variant used in this paper). These structures correspond to anti-parallel dimers. The rhodopsin monomers in each dimer are shown in blue and salmon (PDB 1GZM), and purple and cyan (PDB 2J4Y). Residues V209 and F220 are shown as CPK representations in green and yellow, respectively.

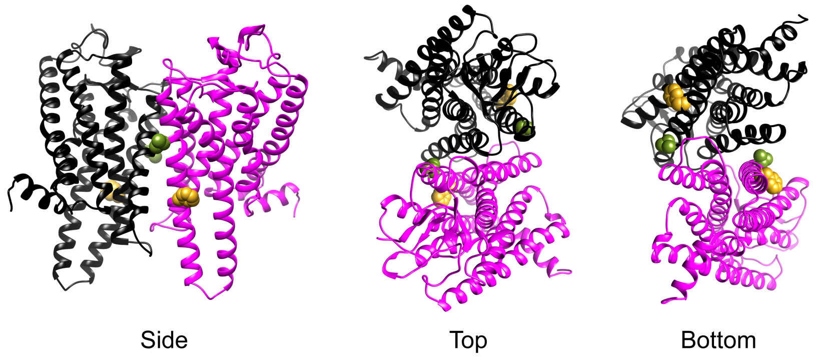

Supplementary Figure 8

Model of an opsin dimer with a TM4/TM5-TM4/TM5 interface. Opsin monomers (PDB 4J4Q) were docked using the HADDOCK web server by applying constraints to residues in TM3 and TM4. The opsin monomers are shown in black and magenta. Residues V209 and F220 are shown as CPK representations in green and yellow, respectively. The top and bottom views correspond to the exoplasmic and cytoplasmic side of the dimer, respectively.

Supplementary Figure 9.

The figure is shown on the next page.

A parallel rhodopsin dimer with TM5 at the interface. A. Docking statistics. The HADDOCK web server was used to dock opsin monomers (PDB 4J4Q) by applying constraints to residues in TM5 and TM6. The dimer models obtained were grouped into 10 clusters according to the parameters shown in the bar chart (normalized to the highest value of each parameter amongst the clusters; the clusters are numbered 1-11, but cluster-3 did not have a high enough score to be placed in the top 10). **B. Dimer models.** Representative dimer models (side and top views, monomers in grey and blue) from each of the clusters, presented in order of their score. Clusters 6 and 9 have similar parallel orientations of the constituent monomers and feature the residues of interest (V209 and F220) at the dimer interface (designated dimer interface 2). Cluster 6 had a higher score and was therefore chosen for further discussion in the main paper.

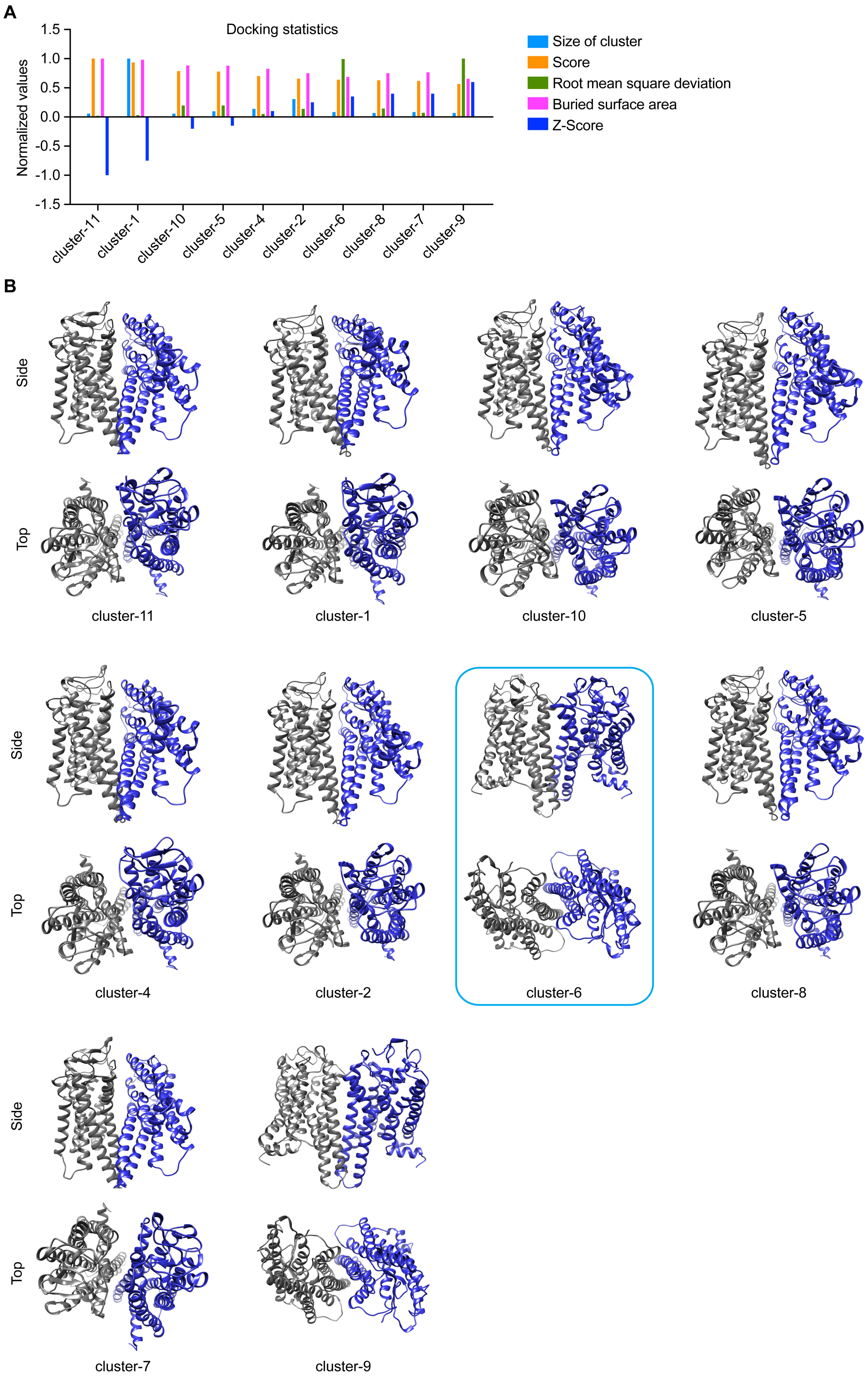
A
Docking statistics
Normalized values
1.5
1.0
0.5
0.0
-0.5
-1.0
-1.5
cluster-11
cluster-1
cluster-10
cluster-5
cluster-4
cluster-2
cluster-6
cluster-8
cluster-7
cluster-9
Size of cluster
Score
Root mean square deviation
Buried surface area
Z-Score
B
Side
Top
cluster-11
cluster-1
cluster-10
cluster-5
cluster-4
cluster-2
cluster-6
cluster-8
cluster-7
cluster-9

Supplementary Tables

Supplementary Table 1

Fit parameters for fluorescence reduction traces

Column #	1	2	3	4
	n	Half-life (sec) (S=free parameter)	Magnitude of line slope S (sec^{-1} x 10^4)	Half-life (sec) (fixed S=0.0001 s^{-1})
No protein	6	14.96 ± 1.07	1.11 ± 0.23	15.48 ± 1.39
Ops (WT)	23	16.44 ± 0.57	1.57 ± 0.16	17.58 ± 0.53
Ops* (M257Y)	12	15.27 ± 0.49	1.63 ± 0.10	16.37 ± 0.56
F45L	10	21.18 ± 1.24	1.15 ± 0.14	20.99 ± 1.18
V209M	10	15.62 ± 0.53	0.73 ± 0.32	15.26 ± 0.59
F220C	8	16.27 ± 0.55	0.78 ± 0.15	15.99 ± 0.65

Fluorescence traces from scramblase assays, including those shown in Supplementary Fig. 2, were analyzed by fitting to the equation F(t)=(1 - Plateau)*exp(-K*t) + Plateau - S*t, where F(t) = fluorescence at time t, t=0 sec is the time of dithionite addition, and S=absolute value of the slope of the linear component. The results are given as mean ± SEM (n= number of independent vesicle reconstitution samples). The standard error of individual fits was at least an order of magnitude lower than the SEM. Values of half-life (= 0.69/K) and S are given in columns 2 and 3 (plateau values are not given as these vary depending on the protein to phospholipid ratio of the sample). The traces were re-analyzed by fixing S=0.0001 s^{-1}; the resulting half-life values obtained are shown in column 4.

Supplementary Table 2

Analysis of p(≥1 scramblase) vs PPR* plots

	α (x 10^4)^	M (g/mol)	~n (Opsin n-mer)$
Ops (WT)	7.44 ± 0.25	96,500 ± 3,240	2 and higher
Ops* (M257Y)	7.91 ± 0.57	90,800 ± 6,540	2
F45L	17.7 ± 1.17	40,600 ± 2,680	1
V209M	15.1 ± 0.95	47,200 ± 2,730	1
F220C	16.2 ± 0.60	44,300 ± 1,640	1

^$\alpha=16/M\varepsilon^2$ is the fit constant in equation 4, with units of $mol.g^{-1}.nm^{-2}$; M is the molar mass of the functional scramblase deduced from the fit, using ε=0.472 nm as the cross-sectional radius of a phospholipid [1]. The standard error associated with the fit is indicated.

#Several combinations of dimer and higher order multimers could account for M~100,000 g/mol for the Ops sample. For example, a mixture of dimers (75%) and tetramers (25%) would yield M~100,000 g/mol. While it is also possible that a fraction of Ops is inactive as a scramblase, resulting in lower α values, we consider this to be unlikely as the protein is fully active *in vitro* as a GPCR (Figure 3, main paper) and no aspect of our protein expression/purification procedure results in inactivation as can be seen from the fact that each of the three RP Mutants reconstitutes as a monomer, i.e. with close to the highest α value possible.

$The molar mass of an opsin monomer is 41,700 g/mol.

Supplementary Notes

Supplementary Note 1

Scramblase activity assay (analysis of fluorescence traces). To measure lipid scrambling, large unilamellar vesicles (LUVs) are symmetrically reconstituted with a trace quantity of fluorescent NBD-phospholipid. The vesicles are treated with dithionite, a membrane-impermeant reagent that irreversibly eliminates the fluorescence of all NBD-phospholipids in the outer leaflet [2]. As spontaneous translocation of phospholipids is not detectable on the time scale (<10 min) of our experiments [3], dithionite treatment of protein-free LUVs is predicted to result in 50% reduction in fluorescence. For samples where every LUV has a scramblase, the predicted reduction in fluorescence on dithionite treatment is 100% as NBD-phospholipids can exchange between the inner and outer leaflet because of scramblase activity. Supplementary Fig. 2 shows examples of fluorescence reduction traces for vesicles reconstituted with different amounts of wild-type (WT) opsin, Ops* (a conformationally distinct state of the protein resulting from the point mutation M257Y) [4], and the three RP-associated opsin mutants.

We previously used a double-exponential function to fit the fluorescence reduction traces and found that the slow component of the fit was typically more than an order of magnitude slower than the fast component and represented a minor fraction of the total fluorescence change [4]. To fit the traces more reliably by reducing the number of fitting parameters, we now approximate the slow component as a variable or constant line as others have done previously [2], thereby reducing the number of fitting parameters from 4 (for a double-exponential) to 3 or 2 (for a mono-exponential with a variable or fixed line, respectively). We find that all fluorescence reduction traces are well described by a combination of a mono-exponential decay function with a half-life of ~15 sec, and a linear component with a slow decay rate (Supplementary Table 1, columns 2 and 3). The half-life of the exponential function was essentially unchanged when we performed the fit using a constant line with a negative slope = 0.0001 sec^{-1} in place of a variable line (Supplementary Table 1, compare columns 4 and 2).

The exponential decay is the main feature of the traces and it describes the dithionite reduction reaction. The half-life of the exponential decay is the same for protein-free liposomes and scramblase-active proteoliposomes, indicating that the dithionite reaction is the rate-limiting step of the assay. Thus, it is possible only to provide a lower limit for the rate of lipid scrambling. For 176-nm diameter vesicles (Supplementary Fig. 4) that contain ~280,000 phospholipids and a single functional opsin scramblase, this indicates a transport rate of >10,000 lipids per second per scramblase. The molecular basis for the slow linear decay component of the fit is not known, but it is unlikely to be due to leakage of dithionite into the vesicles as NBD-glucose trapped within the vesicle lumen is stably protected from dithionite [4]. As the linear decay component is the same for both protein-free liposomes and proteoliposomes, it is clearly not related to the scrambling process, and will not be considered further.

The half-life of fluorescence decay upon dithionite addition was slightly but significantly larger for vesicles reconstituted with the F45L protein compared with vesicles reconstituted with the other opsin variants (Supplementary Table 1 and Supplementary

Fig. 3). While it is not possible to decompose reliably the F45L traces to identify the fast and slow rate processes, this result nevertheless suggests that scrambling by F45L opsin may be impaired such that it occurs on a similar time scale as the dithionite reduction reaction.

Supplementary Note 2

Scramblase activity assay (protein dependence). The scramblase assay reports the extent of fluorescence reduction. For protein-free liposomes, the extent of reduction is 45.0 ± 0.8 % (mean ± SEM, n=6), identical to the value of 46.7 ± 0.9% that we reported previously [4], and close to the expected value of 50% for LUVs that are symmetrically labeled with NBD-phospholipids.

For proteoliposomes, the extent of reduction depends on the protein to phospholipid ratio (PPR) of the sample as the amount of protein used for reconstitution dictates whether an individual vesicle in the sample will contain a functional scramblase. The maximum extent of reduction is expected to be 100%, corresponding to high PPR values where every vesicle should be equipped with a scramblase. However, we obtained a maximum of 82.5 ± 0.5% (n=14), identical to the result that we reported previously (82.6 ± 0.5%) [4]. This indicates that a fraction of the vesicles is *consistently* refractory to reconstitution, an observation that has been made by us and by others in a variety of proteoliposome reconstitution experiments [4-9]. The origin of the refractory population is not known, but as it corresponds to a highly reproducible fraction of the vesicles it may be an intrinsic property of the system. The refractory pool corresponds to 35% of the vesicle population, as 17.5% of the NBD-phospholipid fluorescence is inaccessible to dithionite.

The extent of fluorescence reduction exceeding the value obtained for protein-free liposomes is a measure of the fraction of vesicles that contains at least one functional scramblase. Assuming that opsin reconstitutes randomly into vesicles, this fraction (or equivalently p(≥1 scramblase), the probability that a particular vesicle in the ensemble possesses at least one functional scramblase) can be calculated using Poisson statistics. We previously described a simple version of this calculation, in which we did not consider the refractory pool and assumed that all vesicles were spheres with the same radius.

We now present an advanced calculation that takes into account the size distribution of the vesicles (Supplementary Fig. 4) and uses PPR* rather than the measured PPR to account for the refractory pool. Similar calculations have been described previously [10].

Supplementary Note 3

Analysis of the functional reconstitution of scramblases. We obtained an analytical expression for p(≥1 scramblase) in terms of PPR* as follows. This expression was used to fit the experimental data enabling calculation of the molar mass of the functionally reconstituted scramblase.

1. We define μ = average number of functional scramblases per vesicle, i.e., μ = p/L, where p = total number of functional scramblases and L = total number of vesicles. Assuming that opsin reconstitutes randomly into vesicles, the fraction of vesicles that has zero functional scramblases is $f = e^{-\mu} = e^{-p/L}$ based on Poisson statistics. As the vesicles are not uniform in size, we note that for the subset of L_j vesicles of radius r_j, $f_j = e^{-\mu_j} = e^{-p_j/L_j}$.

2. Assuming that the probability of a scramblase inserting into a vesicle is proportional to the relative surface area of that vesicle, i.e. surface area of the vesicle ÷ total surface area of all vesicles [10], then for the subset of L_j vesicles of radius r_j, $p_j = p\bullet(4\pi r_j^2\bullet L_j)/A$. Thus, $\mu_j = p_j/L_j = 4\pi r_j^2(p/A) = 4\pi r_j^2 z$, where A is the total surface area of all vesicles in the sample and z=p/A.

3. Using the information from steps 1 and 2, and summing over all vesicles, we can write p(≥1 scramblase) = 1 – $\Sigma w_j f_j/\Sigma w_j$, where w_j is a weighting factor and j goes from 1 to N (the total number of vesicles). Assuming a Gaussian frequency distribution of vesicle sizes, with a mean radius $\bar{r}$ and standard deviation σ (Supplementary Fig. 4), and integrating from -∞ to +∞ (permissible when the minimum and maximum r values are much smaller and larger, respectively, than $\bar{r}$), this expression can be written as

$$p(\geq 1\, scramblase) = 1 - \frac{1}{\sqrt{2\pi\sigma^2}}\int_{-\infty}^{\infty} e^{-\frac{(r-\bar{r})^2}{2\sigma^2}} \bullet e^{-4\pi r^2 z}\,dr \quad \text{(Equation 1)}$$

which on integration yields

$$p(\geq 1\, scramblase) = 1 - \frac{1}{\sqrt{1+8\pi\sigma^2 z}} \bullet e^{\frac{-4\pi\bar{r}^2 z}{1+8\pi\sigma^2 z}} \quad \text{(Equation 2)}$$

4. The term z is related to PPR as follows: (i) PPR = m/λ, where m is the mass of reconstituted protein in grams and λ is the amount of lipid in moles; (ii) $p = mN_A/M$, where N_A is Avogadro's number, and M is the molar mass of the functional scramblase; (iii) $A = \lambda N_A \pi\varepsilon^2/2$, where ε is the cross-sectional radius of a phospholipid in nm, and the factor 2 in the denominator is necessary because only half of the total lipids contribute to the outer surface of the vesicles; (iv) Thus, $z = p/A = PPR\bullet(2/M\pi\varepsilon^2)$.

5. Substituting the expression for z into equation 2, and defining $\alpha=16/M\varepsilon^2$ and x=PPR, we obtain

$$p(\geq 1\, scramblase) = 1 - \frac{1}{\sqrt{1+\alpha\sigma^2 x}} \bullet e^{\frac{-\alpha\bar{r}^2 x/2}{1+\alpha\sigma^2 x}} \quad \text{(Equation 3)}$$

6. We used dynamic light scattering to measure the size distribution of the vesicles. Fitting the light scattering data to a Gaussian distribution yielded a mean radius $\bar{r}$ = 88 nm with a standard deviation σ = 28 nm (Supplementary Fig. 4).

7. Using $\bar{r}$ = 88 nm, σ = 28 nm and $\varepsilon = 0.47\ \text{nm}^{1}$, equation 3 can be written as

$$p(\geq 1\,scramblase) = 1 - \frac{1}{\sqrt{1+784\alpha x}} \bullet e^{\frac{-3872\alpha x}{1+784\alpha x}} \quad \text{(Equation 4)}$$

8. We obtained p(≥1 scramblase) by transforming end-point fluorescence reduction data from scramblase activity assays as follows: p(≥1 scramblase) = $(F - F_o)/(F_{max} - F_o)$, where F is the percentage fluorescence reduction for a particular sample 400 s after adding dithionite, F_o is the percentage reduction obtained with protein-free liposomes (~45%, see above) and F_{max} is the maximum percentage reduction observed at high PPR values where all vesicles are expected to have at least one functional scramblase (~82.5%, see above). We also scaled our measured PPR values by a factor of 0.65 to account for the pool of vesicles that is refractory to reconstitution (the fraction of refractory vesicles is 0.35; see above); thus, x = PPR* = (measured PPR)÷0.65.

9. Analysis of the data for Ops (WT) and Ops* (M257Y). Experimental data and fits are shown in Supplementary Fig. 5, and the corresponding fit constant α and the deduced molar mass of the functional scramblase are presented in Supplementary Table 2. Both proteins reconstitute minimally and predominantly as dimers (predicted molar mass of 83,700 g/mol). Data fitting (Supplementary Table 2) reveals a somewhat larger reconstituted unit for Ops(WT) compared with Ops*, consistent with the possibility that Ops (WT) may partly multimerize.

Supplementary Note 4

Pull-down assay. We used WT opsin constructs to identify conditions suitable for pull-down assays. Our initial approach is illustrated in Supplementary Fig. 6A. A mixture of WT Ops-FG and Ops-FG-SNAP in DDM (Supplementary Fig. 6B, Load; see Figure 5A of the main paper for schematic illustration of the constructs) was treated with sufficient BioBeads to reduce but not eliminate detergent [11]; a mock-treated sample was processed alongside. BioBead treatment alone did not affect recovery of the opsin constructs indicating that the proteins were still soluble and had not aggregated or precipitated as a result of reduction in detergent level (Supplementary Fig. 6B, compare lanes 1 and 2). The sample was then incubated with SNAP capture resin. After removing the supernatant, the resin was washed and non-covalently associated proteins were eluted with SDS-containing buffer and analyzed via Coomassie-stained SDS-PAGE. No proteins were detected in the mock-treated sample (Supplementary Fig. 6B, lane 3), consistent with previous data indicating that opsin is monomeric in high levels of DDM [11,12]. However, both Ops-FG and Ops-FG-SNAP were pulled down in the BioBead-treated sample (Supplementary Fig. 6B, compare lanes 3 and 4), indicating dimerization and/or multimerization of opsins when detergent levels are reduced. Pull-down of Ops-FG-SNAP that could be released by SDS is due to homo-dimerization of Ops-FG-SNAP proteins such that only one protomer is covalently bound to SNAP capture resin.

Supplementary Note 5

Modeling opsin dimers with TM5 at the interface. The only explicit structural information on dimers where TM5 is located at or near the dimer interface comes from crystal structures of rhodopsin in which the protomers are in an anti-parallel arrangement (PDB 1GZM and 2J4Y, Supplementary Fig. 7). To generate a more physiologically appropriate model of rhodopsin dimers in which TM5 is positioned at the interaction interface of protomers oriented in parallel, we carried out docking experiments with the HADDOCK webserver [13]. We used the TINKER molecular modeling software (http://dasher.wustl.edu/tinker/) and the OPLS (all atoms) force field [14] to energy-minimize the opsin structure PDB 4J4Q that we used for docking . A HADDOCK-based docking experiment requires that certain residues in the interacting components are designated as active (residues that are directly involved in the interaction under study) while others are considered passive (residues that could potentially be involved in the interaction interface). We picked active and passive residues with the goal of obtaining docked states where (i) TM5 contributes to the interaction interface and (ii) the opsin monomers are aligned parallel to one another.

We carried out two docking experiments, using different sets of active and passive residues. For the first experiment, we considered a dimer interface involving contributions from TM4 and TM5 of each monomer. To this end, residues from the portion of TM3 adjacent to cytoplasmic loop 2 were considered active, whereas residues from TM4 were considered passive (Supplementary Fig. 8). For the second experiment, we considered an interface involving TM5 and TM6, and defined active and passive residues as follows: residues from both TM5 and TM6 adjacent to cytoplasmic loop 3 were considered active whereas those from TM5 adjacent to exoplasmic loop 2 and those from TM6 adjacent to exoplasmic loop 3 were considered passive. This docking experiment also yielded opsin dimers in which TM5 was located at the dimer interface (Supplementary Fig. 9).

Note: We did not assign V209 and F220 as active or passive in either of the docking experiments. All other parameters were the default values provided by HADDOCK. Structural comparisons were done with the matchmaker module of UCSF-Chimera software [15]. Figures were created using UCSF Chimera.

Supplementary References

1 Petrache, H. I., Dodd, S. W. & Brown, M. F. Area per lipid and acyl length distributions in fluid phosphatidylcholines determined by (2)H NMR spectroscopy. *Biophys J* **79**, 3172-3192, (2000).

2 McIntyre, J. C. & Sleight, R. G. Fluorescence assay for phospholipid membrane asymmetry. *Biochemistry* **30**, 11819-11827, (1991).

3 Chalat, M., Menon, I., Turan, Z. & Menon, A. K. Reconstitution of glucosylceramide flip-flop across endoplasmic reticulum: implications for mechanism of glycosphingolipid biosynthesis. *The Journal of biological chemistry* **287**, 15523-15532, (2012).

4 Goren, M. A. *et al.* Constitutive phospholipid scramblase activity of a G protein-coupled receptor. *Nature Communications* **5**, 5115, (2014).

5 Eytan, G. D. Use of liposomes for reconstitution of biological functions. *Biochim Biophys Acta* **694**, 185-202, (1982).

6 Goldberg, A. F. & Miller, C. Solubilization and functional reconstitution of a chloride channel from Torpedo californica electroplax. *J Membr Biol* **124**, 199-206, (1991).

7 Heginbotham, L., Kolmakova-Partensky, L. & Miller, C. Functional reconstitution of a prokaryotic K+ channel. *J Gen Physiol* **111**, 741-749, (1998).

8 Lee, S.-Y., Letts, J. A. & MacKinnon, R. Functional reconstitution of purified human Hv1 H+ channels. *Journal of molecular biology* **387**, 1055-1060, (2009).

9 Malvezzi, M. *et al.* Ca2+-dependent phospholipid scrambling by a reconstituted TMEM16 ion channel. *Nature communications* **4**, 2367, (2013).

10 Walden, M. *et al.* Uncoupling and turnover in a Cl-/H+ exchange transporter. *J Gen Physiol* **129**, 317-329, (2007).

11 Goren, M. A. *et al.* Constitutive phospholipid scramblase activity of a G protein-coupled receptor. *Nat Commun* **5**, 5115, (2014).

12 Ernst, O. P., Gramse, V., Kolbe, M., Hofmann, K. P. & Heck, M. Monomeric G protein-coupled receptor rhodopsin in solution activates its G protein transducin at the diffusion limit. *Proc Natl Acad Sci U S A* **104**, 10859-10864, (2007).

13 de Vries, S. J., van Dijk, M. & Bonvin, A. M. J. J. The HADDOCK web server for data-driven biomolecular docking. *Nature Protocols* **5**, 883-897, (2010).

14 Jorgensen, W. L. & Julian, T.-R. The OPLS (optimized potentials for liquid simulations) potential functions for proteins, energy minimizations for crystals of cyclic peptides and crambin. *J. Am. Chem. Soc.* **110**, 1657-1666, (1988).

15 Pettersen, E. F. *et al.* UCSF Chimera--a visualization system for exploratory research and analysis. *Journal of Computational Chemistry* **25**, 1605-1612, (2004).
